# Validation of a Quick Flow Cytometry-Based Assay for Acute Infection Based on CD64 and CD169 Expression. New Tools for Early Diagnosis in COVID-19 Pandemic

**DOI:** 10.3389/fmed.2021.655785

**Published:** 2021-03-23

**Authors:** Alejandra Comins-Boo, Maria Gutiérrez-Larrañaga, Adriel Roa-Bautista, Sandra Guiral Foz, Mónica Renuncio García, Elena González López, Juan Irure Ventura, María Carmen Fariñas-Álvarez, David San Segundo, Marcos López Hoyos

**Affiliations:** ^1^Immunology Unit, Marqués de Valdecilla University Hospital, Santander, Spain; ^2^Autoimmunity and Transplantation Research Group, Research Institute “Marqués de Valdecilla” (IDIVAL), Santander, Spain; ^3^Infectious Diseases Unit, Marqués de Valdecilla University Hospital, Santander, Spain; ^4^Epidemiology and Pathogenic Mechanisms of Infectious Diseases Research Group, Research Institute “Marqués de Valdecilla” (IDIVAL), Santander, Spain

**Keywords:** biomarkers, flow cytometry, validation, CD169, CD64, SARS-CoV-2

## Abstract

**Objectives:** Several parameters aid in deciphering between viral and bacterial infections; however, new tools should be investigated in order to reduce the time to results and proceed with an early target-therapy. Validation of a biomarker study, including CD64 and CD169 expression, was conducted.

**Material and Methods:** Patients with active SARS-CoV-2 infection (ACov-2), bacterial infection (ABI), healthy controls, and antiretroviral-controlled chronic HIV infection were assessed. Whole blood was stained and, after lysing no-wash protocol, acquired by flow cytometry. The median fluorescence intensity (MFI) of CD64 and CD169 was measured in granulocytes, monocytes, and lymphocytes. The CD64 MFI ratio granulocytes to lymphocytes (CD64N) and CD169 MFI ratio monocytes to lymphocytes (CD169Mo) were evaluated as biomarkers of acute bacterial and viral infection, respectively.

**Results:** A CD64N ratio higher than 3.3 identified patients with ABI with 83.3 and 85.9% sensitivity and specificity, with an area under the curve (AUC) of 83.5%. In contrast, other analytic or hematological parameters used in the clinic had lower AUC compared with the CD64N ratio. Moreover, a CD169Mo ratio higher than 3.3 was able to identify ACov-2 with 91.7 and 89.8 sensitivity and specificity, with the highest AUC (92.0%).

**Conclusion:** This work confirms the previous data of CD64N and CD169Mo ratios in an independent cohort, including controlled chronic viral HIV infection patients as biomarkers of acute bacterial and viral infections, respectively. Such an approach would benefit from quick pathogen identification for a direct-therapy with a clear application in different Health Care Units, especially during this COVID pandemic.

## Introduction

The Siglec-1 or sialoadhesin (CD169) is constitutively expressed on macrophages and has been associated with anti-viral (1) and anti-tumor responses (2–4) and with regulatory function (5). The CD169 ligand is modified-sialic acid and has been involved in removing exosomes by subcapsular macrophages in lymph nodes (6). The expression of CD169 on monocytes is induced after the type-I interferon (IFN) treatment *in vitro* (7).

On the other side, the high-affinity Fc-IgG receptor (CD64) is expressed upon activation on neutrophils, macrophages, and some dendritic cell subsets (8), with different effector functions as opsonization and antibody-dependent cellular cytotoxicity (9). The IFN-gamma induces the CD64 expression on neutrophils *in vitro*, driving to a cellular immune response (7). The immune response against viral pathogens is based on recognizing both viral peptides and viral nucleic acids not present in the host. The receptors involved are Toll-like receptors, the retinoic acid-inducible gene I (RIG-I) receptor protein family, and cytoplasmic DNA receptors, with convergent pathways producing IL-1beta and type I-IFNs (IFN-alpha and IFN-beta, with anti-viral activity. By contrast, the anti-bacterial response induces a cellular response in which one of the main soluble factors is IFN-gamma.

Both CD markers were able to discern an acute bacterial from acute viral infection (10). Recently, an increased expression of CD169 on monocytes was confirmed in acute SARS-CoV-2 infection (11) that remained increased after 2 weeks from symptoms onset.

At present, in the context of the COVID-19 pandemic, one of the main problems is identifying those patients promptly with acute COVID-19 from other causes of infection, especially at admission into the hospital. Based on it, we propose the implementation of quick markers as those based on flow cytometry to classify patients. The present work aimed to validate the potential usefulness of these biomarkers in acute infectious processes, evaluate the ability to discern between acute and chronic viral infections, and assess their utility after a positive PCR to SARS-CoV-2 with time.

## Materials and Methods

A total of 83 samples from patients and healthy subjects were recruited at Marqués de Valdecilla Hospital in November 2020 after informed consent is given. The study was assessed by the regional ethic committee (CEIC, code 2020.167). Previously, the utility of the CD64 and CD169 expression to differentiate between bacterial and viral infection at the emergency unit has been shown (12). In order to validate the assay in an independent cohort, several groups were established: firstly, a group of admitted patients with acute infection was recruited for the study, 12 with active bacterial infection (ABI) with confirmed bacterial pathogen isolation or recovery after antibiotic treatments and 24 patients with active SARS-CoV-2 infection (ACov-2), all the patients included in the group were tested for specific-SARS-CoV-2 polymerase chain reaction (PCR) prior admission; secondly, a group of 18 patients followed in the Infectious Disease Unit with antiretroviral-controlled chronic HIV infection (all of them without viral load at the moment of the assay) and finally, a group of healthy controls (HC) without evidence of infection were included (*n* = 29). The clinical and laboratory findings in each group are summarized in Table 1. The selection of HIV-infected patients with controlled infection with antiretrovirals was to determine the test's ability to discern between acute vs. controlled chronic viral infection.

**Table 1:** Demographic and analytical parameters in the different groups.

	**HC (*n* = 29)**	ABI^*^ (*n* = 12)	**HIV (*n* = 18)**	ACov-2^*^ (*n* = 24)	***p*-value**
**Patient sex**					
Women	16 (55.2%)	8 (66.7%)	1 (5.56%)	12 (50%)	
Age (years)	60 (38–79)	81 (67–90.5)	54 (44–59)	84.5 (63.5–88.5)	*p* < 0.001
**Biochemical parameters**					
CRP (mg/dL)	0.4 (0.4–0.9)	11.9 (5.8–17)	0.4 (0.4–0.4)	5.4 (2.8–10.9)	*p* < 0.001
Ferritin (mg/dL)	202 (119–366)	330 (153–526)	317 (221–413)	687 (260–1,132)	NS (*p* = 0.067)
**Hematological parameters**					
Lymphocyte frequency (%)	23.6 (17.5–32.9)	13.4 (11.7–16.1)	38.7 (28–41.7)	13.8 (7.1–22.9)	*p* < 0.001
Neutrophil frequency (%)	64.2 (52.9–69.5)	78.3 (73.6–82.9)	50.6 (47.1–58.4)	77.7 (67.7–86.5)	*p* < 0.001
Monocyte frequency (%)	8.8 (7.5–11.7)	6.4 (4.9–8.0)	8.7 (7.9–9.7)	7.6 (4.1–9.9)	*p* < 0.01
Lymphocyte count (10^3^ cells/ml)	1.4 (1.1–1.6)	1.2 (0.8–1.8)	1.9 (1.5–2.6)	0.8 (0.5–1.4)	*p* < 0.001
Neutrophil count (10^3^ cells/ml)	3.3 (2.4–6.2)	5.7 (5.1–8.5)	3 (2.1–3.4)	3.9 (3.2–7.4)	*p* < 0.01
Monocyte count (10^3^ cells/ml)	0.5 (0.4–0.8)	0.6 (0.4–0.7)	0.5 (0.4–0.6)	0.5 (0.2–0.6)	NS (*p* = 0.320)
**Radiological test**					
Pneumonia	NP	9 (75%)	NP	24 (100%)	
**Microbiological analysis**					
PCR SARS-CoV-2	NP	0 (0%)	NP	24 (100%)	
Legionella	NP	0 (0%)	NP	0 (0%)	
*Streptococcus pneumoniae*	NP	3 (25%)	NP	0 (0%)	
*Staphylococcus lugdunensis*	NP	1 (8.3%)	NP	0 (0%)	
*Enterobacter cloacae*	NP	1 (8.3%)	NP	0 (0%)	
*Pseudomonas aeruginosa*	NP	2 (16.7%)	NP	0 (0%)	
MRSA	NP	1 (8.3%)	NP	0 (0%)	
*Staphylococcus haemolyticus*	NP	1 (8.3%)	NP	0 (0%)	

*The median and interquartile range (IQR) are shown. Kruskal–Wallis test has been used to determine differences between groups*.

*HC, Healthy Controls; ABI, Acute Bacterial Infection; HIV, Human Immunodeficiency Virus; ACov-2, Acute SARS-Cov-2 Infection; CRP, C-Reactive Protein; PCR, Polymerase Chain Reaction; MRSA, Methicillin Resistant Staphylococcus aureus; NS, Not Significant; NP, Not Performed*.

* *ABI and ACov-2 groups details are summarized in Supplementary Tables 1A,B*.

All the samples were treated as potentially infectious following the current national guidelines and standard operating procedures to manage this kind of sample as suggested by World Health Organization (13).

**Figure 1:**
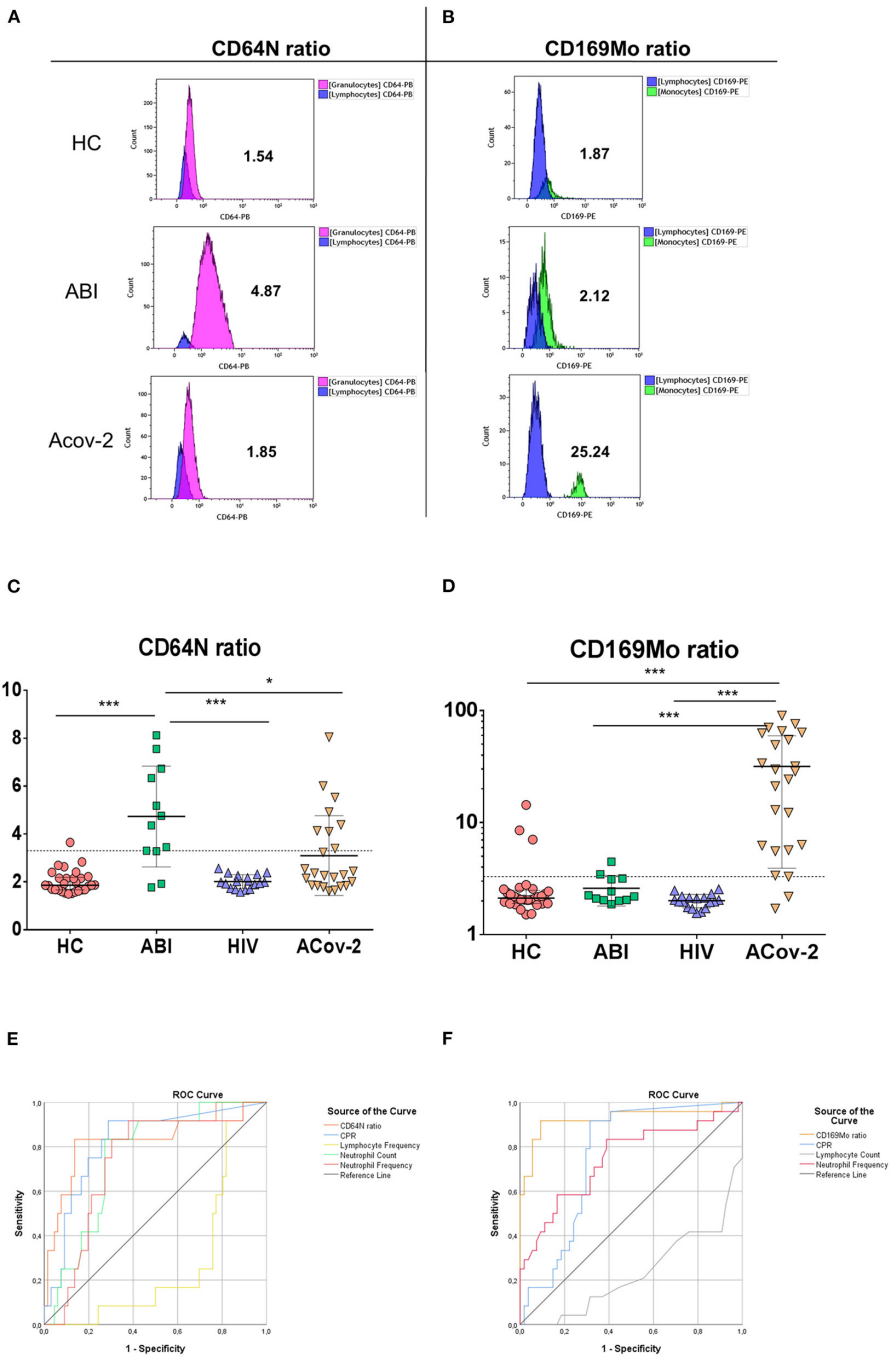
Biomarkers of acute infection. Representative histograms of the median fluorescence intensity in neutrophil granulocytes (purple), lymphocytes (blue), and monocytes (green) of CD64 **(A)** and CD169 **(B)** expression in healthy control (HC), acute bacterial infection (ABI), and acute SARS-CoV-2 infection (ACov2) patient to calculate the ratio CD64 and CD169 as described in Material and Methods section. The ratios of CD64N **(A)** and CD169Mo are depicted **(B)**. The ratio CD64N **(C)** and CD169Mo **(D)** in HC (red light circles), ABI (green squares), chronically HIV infected patients (HIV, blue triangles), and acute SARS-CoV-2 (ACov-2, yellow triangles) are depicted. The dotted lines represent the cut-off value for calculating the Receiver Operational Characteristic (ROC) curve. The ROC curve of different parameters used to decipher acute infections are shown, C-reactive protein (blue line), neutrophil frequency (red line), absolute neutrophil count (green line), lymphocyte frequency (yellow line), lymphocyte count (gray line), CD64N ratio (orange line) **(E)**, and CD169Mo ratio (orange line) **(F)**. ***p <0.001 and *p < 0.05.

One-step flow cytometry staining was performed. Briefly, 25 μL of EDTA whole blood sample was stained with 10 μL of the monoclonal antibody cocktail of CD169-phycoerythrin (PE)/HLA-DR-allophycocyanin (APC)/CD64-pacific blue (PB) and, simultaneously, added 500 μL of lysis buffer Optilyse® using a non-wash protocol (Beckman Coulter Inc, Brea, CA) during 15 min in the dark. The samples were acquired on 10-color flow cytometry (Navios EX) and analyzed by Kaluza Software (Beckman Coulter Inc, Brea, CA). The lymphocytes, monocytes, and neutrophils were gated based on forward and side scatter, and the median events in each population were 4,055, 1,015, and 8,944, respectively (Supplementary Figure 1). The median fluorescence intensity (MFI) in PE (CD169) and PB (CD64) channels was measured in each population. The MFI of CD64 expression on neutrophil to lymphocyte (CD64N) ratio and the MFI of CD169 expression on monocyte to lymphocyte (CD169Mo) ratio was calculated (Figures 1A,B).

### Statistical Analysis

Statistical analysis was performed using SPSS software (Version 25.0, SPSS Inc, Chicago, IL, USA) and GraphPad Prism software version 6.0 (GraphPad Software, La Jolla, CA, USA). Descriptive data are presented as the median and interquartile range (IQR). Qualitative variables were shown as frequencies with percentages, and chi-square was performed to compare the data. The difference between groups was analyzed by Kruskal–Wallis analysis of ranks for non-parametric data following by the Mann–Whitney *U*-test and for parametric data *t*-Student test was used. Receiver operating characteristic (ROC) curves were used to select the optimal cut-off values of both biomarkers that better discriminate bacterial from acute viral infections. The optimal cut-off values were calculated using the Youden Index. Statistical significance was determined when *p* < 0.05 (the significant level was assigned as ^*^p < 0.05, ^**^p < 0.01, ^***^p < 0.001).

## Results

### The CD64N Ratio Is Increased in Acute Bacterial Infection

The median value of CD64N ratio was statistically increased in patients with acute bacterial infection (ABI) 4.56 IQR (3.29–6.53) vs. 1.86 (1.67–2.16) in HC group vs. 1.99 (1.74–2.26) in HIV group vs. 2.33 (1.85–4.11) inACov-2 group (*p* < 0.001, *p* < 0.001, *p* = 0.024), respectively (Figure 1C).

The CD64N ratio was statistically correlated with the different parameters used in clinic routine (Table 2) in the assessment of acute bacterial infections [WBC, frequency of neutrophils and lymphocytes, percentage of immature neutrophils, and C-reactive protein (CRP)] (for more details see Supplementary Table 1A).

**Table 2:** Correlation coefficients and *p*-values between biochemical and hematological parameters and biomarkers of acute infection.

	**CD64N ratio**	**CD169Mo ratio**
CRP	0.565^***^	0.359^**^
Ferritin	0.364^*^	ns
Lymphocyte frequency	−0.546^***^	ns
Neutrophil frequency	0.532^***^	0.223^*^
Monocyte frequency	−0.339^**^	ns
Lymphocyte count	−0.352^**^	−0.259^*^
Neutrophil count	0.431^**^	ns

*** *p < 0.001*,

** p < 0.01 and

* *p < 0.05*.

*CRP, C-Reactive Protein; ns, not significant*.

In order to assess the best parameter to discriminate between acute bacterial infections, a receiver operating characteristic curve (ROC) for each parameter was calculated. Within all parameters studied, CD64N ratio had the highest area under the curve (AUC): 83.5% compared with 81.0, 75.5, 73.0, and 72.0% in C-Reactive protein, neutrophil counts, frequency of neutrophils, and frequency of lymphocytes (Figure 1E). A CD64N ratio of 3.3 was able to detect acute bacterial infection with 83.3 and 85.9% sensitivity and specificity, respectively. Remarkably, seven patients included in the ACov-2 group also had an increased CD64N ratio, and two of them had a confirmed bacterial coinfection.

### The CD169Mo Ratio Is Increased in Acute Viral Infection by SARS-CoV-2

The median value of CD169Mo ratio was statistically increased in patients with acute viral infection 26.41 (5.94–58.65) vs. 2.12 (1.9–2.43) in HC group vs. 2.01 (1.76–2.13) in HIV group vs. 2.22 (2.04–3.14) in ABI group (*p* < 0.001, *p* < 0.001, *p* < 0.001), respectively (Figure 1D). The CD169Mo ratio was significantly correlated with different clinic routine parameters in assessing acute viral infections (Table 2) (For more details, see Supplementary Table 1B).

In order to assess the best cut-off value to discriminate between acute viral infections, the receiver operator characteristic curve (ROC) for each parameter was also calculated. CD169Mo had the highest AUC 92.0%, within all parameters studied compared with 77.0, 75.8, 75.4, and 74.5% of lymphocyte counts, CRP, serum ferritin, and neutrophil frequency, respectively (Figure 1F). A ratio of CD169Mo of 3.3 was able to detect acute viral infection with 91.7 and 89.8 sensitivity and specificity, respectively (details of positive predictive value and negative predictive value and likelihood of all parameters associated with acute infection are summarized in Supplementary Table 2).

**Figure 2:**
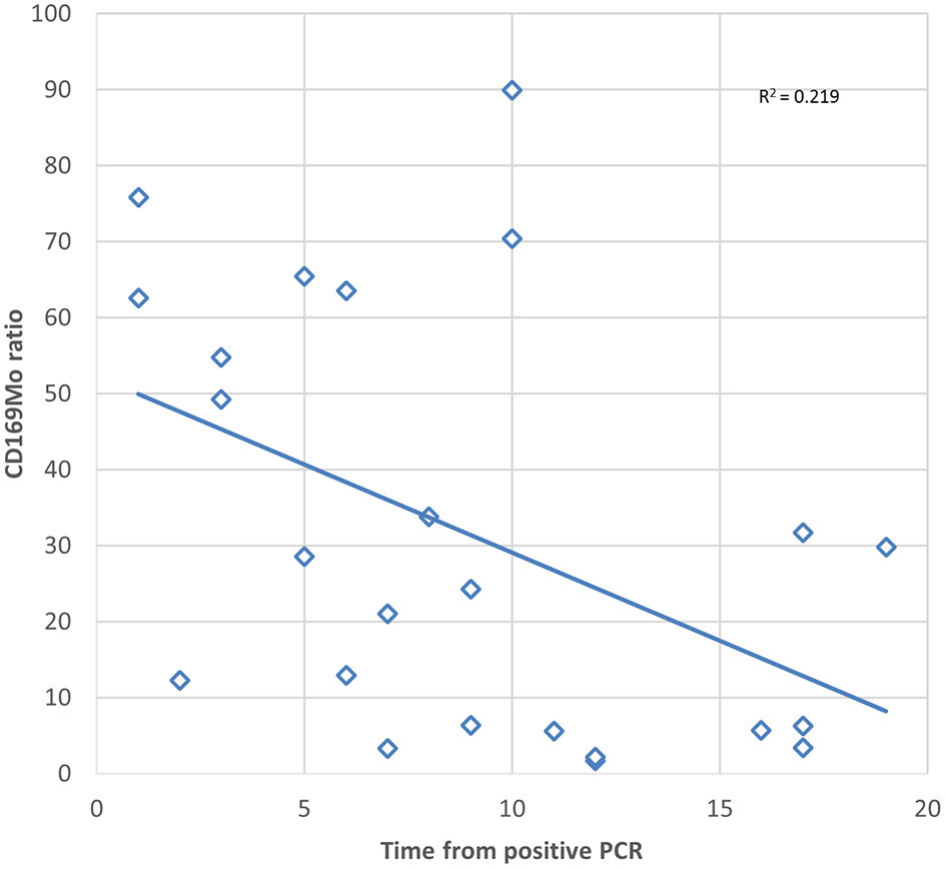
Scatter plot showing the correlation between CD169Mo ratio and time in days from positive PCR of SARS-CoV-2.

In order to assess the duration of the usefulness of the CD169Mo ratio after a positive PCR, a Spearman correlation was tested, and a significant negative association between the CD169Mo ratio and time from positive PCR was observed *p* = 0.021 (Figure 2).

## Discussion

The early identification of patients with acute SARS-CoV-2 infection at hospital admission is becoming of increased interest in order to identify those patients with COVID-19 disease. The PCR remains the gold standard test but with the pitfall of delayed time to results that usually takes no < 4 h. This validation work confirms the one-step flow cytometry-based assay in < 1 h as a suitable test to detect acute viral infection by SARS-CoV-2 and ABI. Both CD64N and CD169Mo ratios were able to detect ACov-2 and ABI better than the current biochemical parameters used in clinical routine to discern between acute viral or bacterial infection. The present work shows a cut-off value of 3.3 in both biomarkers, very similar to that described previously (14). However, this assay is not specific to ACov-2 infection since CD169Mo was increased in acute parainfluenza, human respiratory syncytial, and C-hepatitis virus infections at emergency units (12, 15).

Our validation cohort included a group of chronically infected HIV patients controlled by antiretroviral treatment and without HIV viral load at the assay moment. In this group, both biomarker values were comparable with the observed in the HC group, pointing to the exclusive role of CD169Mo ratio as a marker for acute viral infection, which normalizes after infection chronicity. This assay has the potential to detect acute viral and bacterial coinfection when both CD64N and CD169Mo are increased (12), as observed in two cases of ACov-2 in our cohort.

On the other hand, one patient of the ABI group had a CD64N ratio below the cut-off, and this value could be due to the prolonged time from the first bacterial isolation to the assay (18 days). However, this patient presented several different isolations during admission, with antibiotic treatment that could interfere with the CD64N ratio. In the same direction, two patients included in the ACov-2 group presented a low CD169Mo ratio, and in both cases, the positive PCR was detected 12 days before the assay. This observation fits with the decrease in the CD169Mo ratio after several days from positive PCR. Although this assay is intended for the acute phase of infection, further investigations should point to the dynamic of CD64N and CD169Mo expression with the clearance of pathogens to validate their usefulness in later steps of infections. In this study, a weak correlation was observed between the expression of CD169 from positive PCR, which may be due to lower activation of monocytes together with a greater clearance of the virus. A longitudinal study would be necessary to confirm this correlation.

An increased expression of CD169Mo in anti-tumoral response (16, 17) and children with active systemic lupus erythematosus has been described (18). Therefore, in these patient profiles, the CD169Mo ratio can be skewed and should be considered as potential confounders. In addition, the value of the CD169Mo ratio in patients with another autoimmune disease is still unknown.

These observations strongly suggest that the CD64N and CD169Mo ratio are robust biomarkers of acute infections, but the time after treatment reduces the capability to differentiate from bacterial or viral infection. In the current pandemic era, this assay could be used to identify patients with ACov-2 suspicion, but further SARS-Cov-2 PCR is mandatory to confirm the pathogen involved in CD169Mo expression rise.

A limitation of this study is that the number of patients included with bacterial isolation is scarce. We have included one patient in whom the isolation was confirmed 6 days after the analysis, and 4 patients recovered after antibiotic treatment. For this reason, we did not perform further correlation analysis with the CD64N ratio. Recently, it has been demonstrated the potential role of CD64 expression in patients with sepsis in pediatric intensive care units (19, 20).

In conclusion, this study confirms the previous data of CD64N ratio and CD169Mo in an independent cohort and including controlled chronic viral HIV infection as a potential biomarker able to discern between acute bacterial and viral infections. These biomarkers would have a clear application in different Health Care Units and would benefit from a quick pathogen identification for a direct therapy.

## Data Availability Statement

The raw data supporting the conclusions of this article will be made available by the authors, without undue reservation.

## Ethics Statement

The studies involving human participants were reviewed and approved by CEIC, code 2020.167. The patients/participants provided their written informed consent to participate in this study.

## Author Contributions

DS and ML conceptualization, supervision and review of the draft manuscript. AC-B and DS wrote the manuscript. ML and MCF-A searched for funding. AC-B, DS, and MG-L: statistical analysis. AR-B, SG, MR, EG, and JI: methodology and data management. All authors contributed to the article and approved the submitted version.

## Conflict of Interest

The authors declare that the research was conducted in the absence of any commercial or financial relationships that could be construed as a potential conflict of interest.

## Supplementary Material

Supplementary Figure 1 Gating strategies are displayed. The median (range) of granulocytes events (pink) is 8,944 (1,229–49,726) of monocytes (green) are 1,015 (137–3,562) and 4,055 (7,45–234,450) for lymphocytes (blue).

Supplementary Table 1A Main characteristics of ABI group patients. ID, identification; ABI, Acute Bacterial Infection; PCR, Polymerase Chain Reaction; CRP, C-Reactive Protein; N/L, neutrophils/lymphocytes; Mo/L, monocytes/lymphocytes; RTI, Respiratory Tract Infection; UTI, Urinary Tract Infection.

Supplementary Table 1B Main characteristics of ACov2 group patients. ID, identification; ACov2, Acute SARS-CoV-2 Infection; PCR, Polymerase Chain Reaction; CRP, C-Reactive Protein; N/L, neutrophils/lymphocytes; Mo/L, monocytes/lymphocytes; Severity, mild (without oxygen-therapy), moderate (conventional oxygen therapy) and severe (high-flow nasal cannula oxygenation device).

Supplementary Table 2 Evaluation indexes of diagnostic tools in ABI and ACoV2 groups. CRP, C-Reactive Protein; AUC, area under the curve; PPV, positive predictive value; NPV, negative predictive value; ABI, Acute Bacterial Infection; ACoV2, Acute SARS-CoV-2 Infection; ROC, Receiver Operational Characteristic (ROC). The variables included in Supplementary Table 2 were those included in each ROC analysis (ABI and ACoV2).
