# Supplementary figures and images for: Validation of a Quick Flow Cytometry-Based Assay for Acute Infection Based on CD64 and CD169 Expression. New Tools for Early Diagnosis in COVID-19 Pandemic
Source: Front Med (Lausanne). 2021 Mar 23;8:655785. doi: 10.3389/fmed.2021.655785 (PMC8044950; doi:10.3389/fmed.2021.655785)

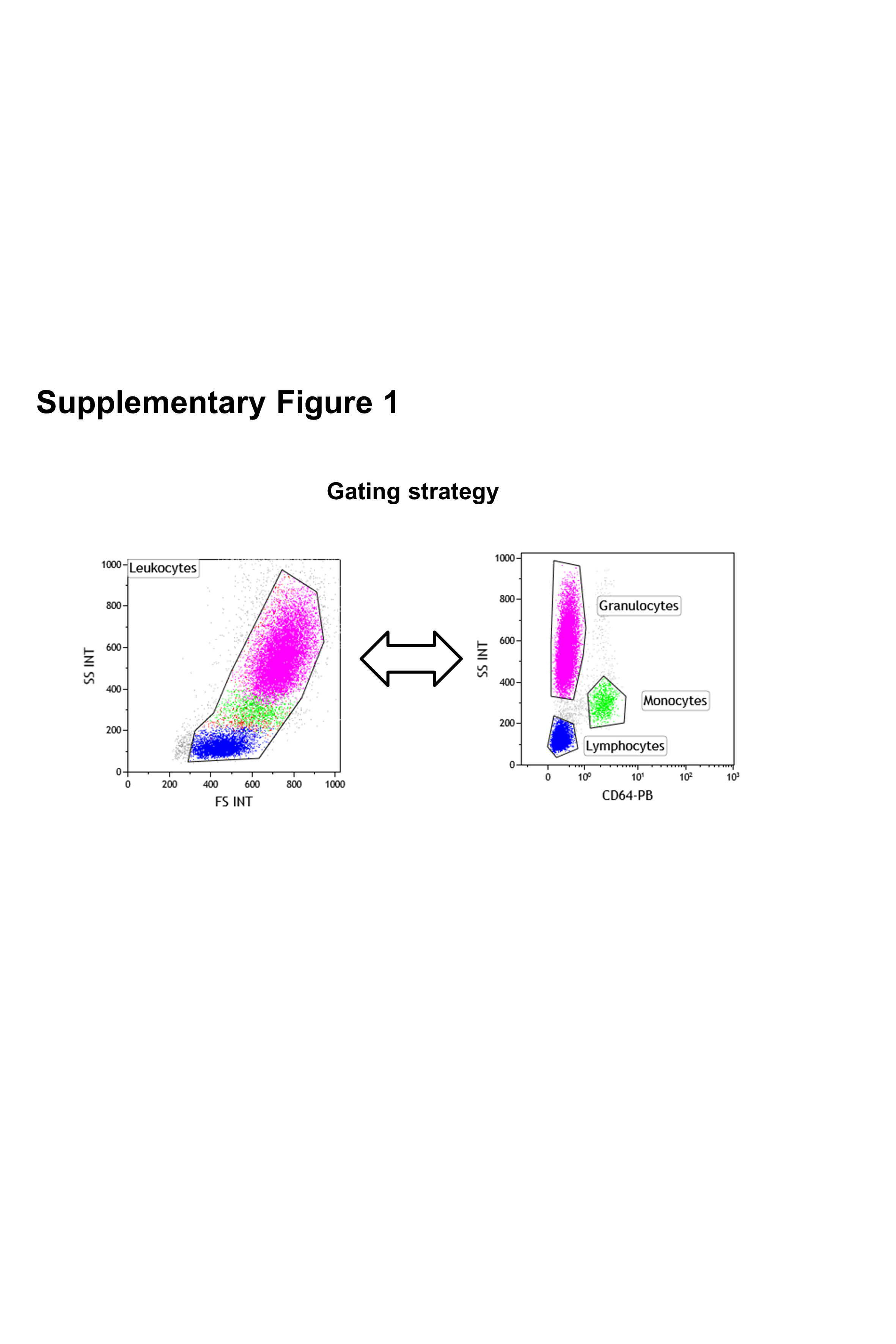

Supplement: Supplementary Figure 1 — Gating strategies are displayed. The median (range) of granulocytes events (pink) is 8,944 (1,229–49,726) of monocytes (green) are 1,015 (137–3,562) and 4,055 (7,45–234,450) for lymphocytes (blue). [file Image_1.TIF]
