# Supplementary material for: Validation of a Quick Flow Cytometry-Based Assay for Acute Infection Based on CD64 and CD169 Expression. New Tools for Early Diagnosis in COVID-19 Pandemic
Source: Front Med (Lausanne). 2021 Mar 23;8:655785. doi: 10.3389/fmed.2021.655785 (PMC8044950; doi:10.3389/fmed.2021.655785)
Supplement: Supplementary Table 1A — Main characteristics of ABI group patients. ID, identification; ABI, Acute Bacterial Infection; PCR, Polymerase Chain Reaction; CRP, C-Reactive Protein; N/L, neutrophils/lymphocytes; Mo/L, monocytes/lymphocytes; RTI, Respiratory Tract Infection; UTI, Urinary Tract Infection. [file Table_1.DOC]

| ID ABI | *Age* | *Date of Analysis* | *PCR neg* | *Date of admission* | *Date of isolation* | *Days from admission to analysis* | *Days from isolation to analysis* | *Date of discharge* | *CRP* | *Ratio CD64 (N/L)* | *Ratio CD169 (Mo/L)* | *Antibiotic treatment* | *Type of infection* | *Pathogen* |
| --- | --- | --- | --- | --- | --- | --- | --- | --- | --- | --- | --- | --- | --- | --- |
| 1 | 68 | 12/03/20 | 12/03/20 | 12/02/20 | 12/03/20 | 1 | 0 | 12/14/20 | 18.2 | 7.55 | 3.17 | Daptomycin | Bacteriemia | Staph.lugdunensis |
| 2 | 66 | 12/03/20 | 12/05/20 | 12/01/20 | 12/03/20 | 2 | 0 | 12/14/20 | 12.9 | 1.77 | 2.03 | Amoxicilin clavulanic > Daptomycin > Meropenem | RTI | Enterocc cloacae |
| 3 | 55 | 12/12/20 | 12/10/20 | 12/11/20 | 12/11/20 | 1 | 1 | 12/15/20 | 14.7 | 8.12 | 3.11 | Levofloxacin > Ceftriasone | RTI | Strep. Pneumoniae |
| 4 | 86 | 12/14/20 | 12/12/20 | 12/13/20 | 12/13/20 | 1 | 1 | 12/16/20 | 8.8 | 3.3 | 2.24 | Levofloxacin | UTI | Strep. Pneumoniae |
| 5 | 96 | 12/04/20 | 12/04/20 | 12/04/20 | - | 0 |  | 12/09/20 | 6.2 | 6.33 | 1.87 | Amoxicilin clavulanic | RTI | NO isolation |
| 6 | 88 | 12/02/20 | 12/01/20 | 12/01/20 | - | 1 |  | 12/04/20 | 24.4 | 6.73 | 2.19 | Amoxicilin clavulanic | RTI | NO isolation |
| 7 | 77 | 12/14/20 | 12/13/20 | 12/13/20 | 12/13/20 | 1 | 1 | 12/17/20 | 0.4 | 1.92 | 3.45 | Carbapemen | UTI | Psuedomonas aereginosa |
| 8 | 85 | 12/12/20 | 12/09/20 | 12/10/20 | 12/10/20 | 2 | 2 | 12/24/20 | 11.0 | 3.28 | 2.43 | Ceftriasone > Clindamycin | RTI | Strep. Pneumoniae |
| 9 | 64 | 12/02/20 | 11/30/20 | 12/01/20 | - | 1 |  | 12/21/20 | 30.4 | 4.76 | 2.04 | Meropenem | Septic shock | NO isolation |
| 10 | 95 | 12/16/20 | 12/15/20 | 12/16/20 | - | 0 |  | 12/18/20 | 15.8 | 4.36 | 4.47 | Azithromycin > Levofloxacin | RTI | NO isolation |
| 11 | 72 | 12/04/20 | 03/12/20 | 12/04/20 | 12/04/20 | 0 | 0 | 12/12/20 | 5.4 | 5.18 | 2.01 | Clindamycin | RTI | St. haemolyticus |
| 12 | 93 | 12/12/20 | 12/10/20 | 12/10/20 | 12/10/20 | 2 | 2 | 12/15/20 | 4.7 | 3.45 | 2.1 | Levofloxacin | Cutaneous infection | Strep. agalactiae + Morganella morganii |

Supplementary Table 1a. Main characteristics of ABI group patients

Abbreviations: ID: identification; ABI: Acute Bacterial Infection; PCR: Polymerase Chain Reaction; CRP: C-Reactive Protein; N/L: neutrophils/lymphocytes; Mo/L: monocytes/lymphocytes; RTI: Respiratory Tract Infection; UTI: Urinary Tract Infection.

*Supplementary Table 1b. Main characteristics of ACov2 group patients*

| *ID ACov2* | *Age* | *Severity* | *Date of Analysis* | *PCR+* | *Date of admission* | *Days from admission to analysis* | *Days from PCR+ to Analysis* | *Date of discharge* | *CRP* | *Ratio CD64 (N/L)* | *Ratio CD169 (Mo/L)* |
| --- | --- | --- | --- | --- | --- | --- | --- | --- | --- | --- | --- |
| *1* | 85 | severe | 12/15/20 | 12/09/20 | 12/10/20 | 5 | 6 | 01/11/21 | 26.0 | 2.36 | 12.94 |
| *2* | 51 | moderate | 12/04/20 | 12/02/20 | 12/02/20 | 2 | 2 | 12/09/20 | 1.1 | 2.54 | 12.21 |
| *3* | 89 | moderate | 12/16/20 | 11/29/20 | 12/15/20 | 1 | 17 | 12/21/20 | 25.3 | 8.04 | 31.71 |
| *4* | 94 | severe | 12/03/20 | 11/21/20 | 11/24/20 | 9 | 12 | 12/11/20 | 9.5 | 2.15 | 1.71 |
| *5* | 85 | mild | 12/04/20 | 11/29/20 | 12/01/20 | 3 | 5 | 12/15/20 | 5.1 | 1.86 | 65.43 |
| *6* | 91 | severe | 12/14/20 | 11/27/20 | 11/30/20 | 14 | 17 | 12/16/20 | 21.0 | 5.52 | 6.22 |
| *7* | 91 | moderate | 12/14/20 | 12/02/20 | 12/12/20 | 2 | 12 | 12/21/20 | 5.3 | 4.91 | 2.18 |
| *8* | 84 | severe | 12/12/20 | 12/02/20 | 12/10/20 | 2 | 10 | 12/17/20 | 12.8 | 4.38 | 89.93 |
| *9* | 70 | severe | 12/03/20 | 11/14/20 | 11/15/20 | 18 | 19 | 12/04/20 | 3.7 | 6 | 29.77 |
| *10* | 54 | moderate | 12/03/20 | 11/24/20 | 11/28/20 | 5 | 9 | 12/03/20 | 1.5 | 1.61 | 6.32 |
| *11* | 88 | moderate | 12/02/20 | 12/01/20 | 12/02/20 | 0 | 1 | 12/09/20 | 5.6 | 1.84 | 75.83 |
| *12* | 97 | moderate | 12/12/20 | 12/02/20 | 12/05/20 | 7 | 10 | 12/16/20 | 5.5 | 4.13 | 70.33 |
| *13* | 87 | moderate | 12/03/20 | 11/30/20 | 11/29/20 | 4 | 3 | 12/10/20 | 5.6 | 2.44 | 54.72 |
| *14* | 72 | mild | 12/04/20 | 11/18/20 | 12/03/20 | 1 | 16 | 12/09/20 | 4.2 | 2.29 | 5.66 |
| *15* | 75 | mild | 12/04/20 | 11/17/20 | 11/27/20 | 7 | 17 | 12/05/20 | 2.8 | 2.19 | 3.37 |
| *16* | 84 | severe | 12/03/20 | 11/26/20 | 11/26/20 | 7 | 7 | 12/12/20 | 23.1 | 3.23 | 21.06 |
| *17* | 69 | mild | 12/16/20 | 12/09/20 | 12/09/20 | 7 | 7 | 12/16/20 | 0.7 | 3.39 | 3.29 |
| *18* | 35 | moderate | 12/02/20 | 11/26/20 | 12/01/20 | 1 | 6 | 12/10/20 | 3.7 | 1.78 | 63.5 |
| *19* | 58 | moderate | 12/01/20 | 11/22/20 | 11/28/20 | 3 | 9 | 12/10/20 | 11.4 | 1.84 | 24.26 |
| *20* | 85 | moderate | 12/04/20 | 12/03/20 | 12/04/20 | 0 | 1 | 12/10/20 | 6.4 | 4.09 | 62.57 |
| *21* | 94 | moderate | 12/12/20 | 12/07/20 | 12/09/20 | 3 | 5 | 12/26/20 | 0.4 | 2.00 | 28.56 |
| *22* | 85 | mild | 12/15/20 | 12/07/20 | 12/14/20 | 1 | 8 | 12/18/20 | 2.7 | 2.26 | 33.76 |
| *23* | 43 | mild | 12/12/20 | 12/09/20 | 12/11/20 | 1 | 3 | 12/16/20 | 1.4 | 1.65 | 49.21 |
| *24* | 44 | mild | 12/18/20 | 12/07/20 | 12/11/20 | 7 | 11 | 12/22/20 | 10.3 | 1.81 | 5.55 |

Abbreviations: ID: identification; ACov2: Acute SARS-CoV-2 Infection; PCR: Polymerase Chain Reaction; CRP: C-Reactive Protein; N/L: neutrophils/lymphocytes; Mo/L: monocytes/lymphocytes; Severity: mild (without oxygen-therapy), moderate (conventional oxygen therapy) and severe (high-flow nasal cannula oxygenation device).
