# Supplementary material for: Validation of a Quick Flow Cytometry-Based Assay for Acute Infection Based on CD64 and CD169 Expression. New Tools for Early Diagnosis in COVID-19 Pandemic
Source: Front Med (Lausanne). 2021 Mar 23;8:655785. doi: 10.3389/fmed.2021.655785 (PMC8044950; doi:10.3389/fmed.2021.655785)
Supplement: Supplementary Table 2 — Evaluation indexes of diagnostic tools in ABI and ACoV2 groups. CRP, C-Reactive Protein; AUC, area under the curve; PPV, positive predictive value; NPV, negative predictive value; ABI, Acute Bacterial Infection; ACoV2, Acute SARS-CoV-2 Infection; ROC, Receiver Operational Characteristic (ROC). The variables included in Supplementary Table 2 were those included in each ROC analysis (ABI and ACoV2). [file Table_2.DOC]

Supplementary Table 2. Evaluation indexes of diagnostic tools in ABI and ACoV2 groups.

|  | **AUC** | **Optimal cut-off value** | **Sensitivity (%)** | **Specificity (%)** | **PPV (%)** | **NPV (%)** | **Positive Likelihood ratio** | **Negative Likelihood ratio** |
| --- | --- | --- | --- | --- | --- | --- | --- | --- |
| **CD169Mo ratio**  **(ACoV2)** | 0.93 | 3.3 | 91.67 | 89.83 | 78.57 | 96.36 | 9.01 | 0.09 |
| **CD64N ratio**  **(ABI)** | 0.84 | 3.3 | 83.33 | 87.32 | 52.63 | 96.88 | 6.57 | 0.19 |
| **CRP (ABI)** | 0.82 | 4.5 | 91.67 | 71.21 | 36.67 | 97.92 | 3.18 | 0.12 |
| **Neutrophils % (ABI)** | 0.74 | 71.3 | 83.33 | 71.01 | 33.33 | 96.08 | 2.88 | 0.23 |
| **Neutrophils Count** (10^3^cells/ml)  **(ABI)** | 0.76 | 4.8 | 83.33 | 73.91 | 35.71 | 96.23 | 3.19 | 0.23 |
| **Lymphocytes %**  **(ABI)** | 0.73 | 18.1 | 83.33 | 71.01 | 33.33 | 96.08 | 2.88 | 0.23 |
| **CRP (ACov2)** | 0.76 | 1.0 | 91.70 | 68.50 | 56.41 | 94.87 | 2.91 | 0.12 |
| **Neutrophils %**  **(ACov2)** | 0.75 | 66.5 | 91.67 | 56.52 | 26.83 | 97.50 | 2.11 | 0.15 |
| **Lymphocytes Count** (10^3^cells/ml)  **(ACoV2)** | 0.77 | 0.9 | 73.68 | 83.87 | 58.33 | 91.23 | 4.57 | 0.31 |

Abbreviations: CRP: C-Reactive Protein; AUC: area under the curve; PPV: positive predictive value; NPV: negative predictive value; ABI: Acute Bacterial Infection; ACoV2: Acute SARS-CoV-2 Infection; ROC: Receiver Operational Characteristic (ROC)

The variables included in Supplementary Table 2 were those included in each ROC analysis (ABI and ACoV2).
